# A Novel Hyaluronic Acid Matrix Ingredient with Regenerative, Anti-Aging and Antioxidant Capacity

**DOI:** 10.3390/ijms24054774

**Published:** 2023-03-01

**Authors:** Patricia Galvez-Martin, Cristina Soto-Fernandez, Jessica Romero-Rueda, Jesus Cabañas, Anna Torrent, Gloria Castells, Daniel Martinez-Puig

**Affiliations:** 1 R&D Animal and Human Health, Bioibérica S.A.U., E-08029 Barcelona, Spain; 2 Health & Biomedicine Department, Leitat Technological Centre, E-08028 Barcelona, Spain; 3 Pharmacy Analysis Service, Department of Pharmacology, Therapeutics and Toxicology, Faculty of Veterinary, E-08193 Bellaterra, Spain

**Keywords:** hyaluronic acid, glycosaminoglycans, collagen, anti-aging, antioxidant, skin

## Abstract

Hyaluronic acid (HA) and proteoglycans (such as dermatan sulphate (DS) and chondroitin sulphate (CS)) are the main components of the extracellular matrix of the skin, along with collagen and elastin. These components decrease with age, which implies a loss of skin moisture causing wrinkles, sagging and aging. Currently, the external and internal administration of effective ingredients that can reach the epidermis and dermis is the main alternative for combating skin aging. The objective of this work was to extract, characterise and evaluate the potential of an HA matrix ingredient to support anti-aging. The HA matrix was isolated and purified from rooster comb and characterised physicochemically and molecularly. In addition, its regenerative, anti-aging and antioxidant potential and intestinal absorption were evaluated. The results show that the HA matrix is composed of 67% HA, with an average molecular weight of 1.3 MDa; 12% sulphated glycosaminoglycans, including DS and CS; 17% protein, including collagen (10.4%); and water. The in vitro evaluation of the HA matrix’s biological activity showed regenerative properties in both fibroblasts and keratinocytes, as well as moisturising, anti-aging and antioxidant effects. Furthermore, the results suggest that the HA matrix could be absorbed in the intestine, implying a potential oral as well as topical use for skin care, either as an ingredient in a nutraceutical or a cosmetic product.

## 1. Introduction

The skin is the largest organ of the body, representing one-sixth of the total body weight with an approximate surface area of 1.5–2 m [1,2]. Its primary role is to provide a physical barrier to protect internal tissues from the external environment and it is, therefore, constantly exposed to mechanical and chemical threats, temperature changes, pathogens, ultraviolet radiation and dehydration [3].

**Figure 1:**
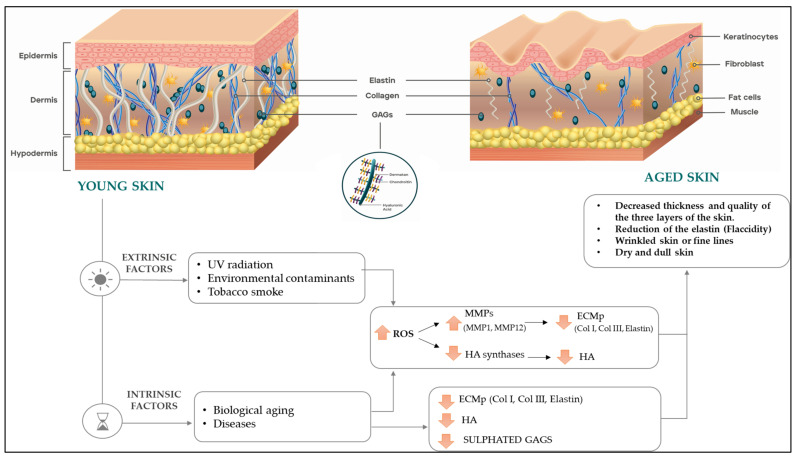
Changes in skin structure and composition due to the aging process. Abbreviations: UV (ultraviolet); ROS (reactive oxygen species); MMP (metalloproteinases); HA (hyaluronic acid); ECMp (extracellular matrix proteins); Col (collagen); GAGs (glycosaminoglycans).

The skin consists of three layers called the epidermis, dermis and hypodermis, each with different structures and compositions (Figure 1). The epidermis is the most superficial layer whose function is to prevent both water loss from the body and penetration of pathogens from the environment. Structurally, the epidermis is made up of dense cell layers, mainly keratinocytes, which synthesise keratin, phospholipids and sphingolipids and critically contribute to the barrier function [4]. In contrast, fibroblasts are the most abundant cells in the dermis. They are responsible for synthesising and remodelling a complex extracellular matrix (ECM) containing collagen, elastin and glycosaminoglycans (GAGs) [5]. Collagen fibres make up about 75% of the dry weight of the skin and they provide tensile strength [6], while elastin forms a network with collagen fibres and provides elasticity [5]. GAGs, mainly composed of hyaluronic acid (HA), dermatan sulphate (DS) and chondroitin sulphate (CS), have both structural and signalling roles. Structurally, GAGs help maintain skin hydration, and from a signalling perspective, they regulate cell migration, proliferation and differentiation, as well as ECM homeostasis [7]. In addition, their potential to moderate cytokines and antioxidant status in animals has been described [8]. Below the dermis, the hypodermis is composed of adipose and connective tissue and provides insulation from heat and cold [9].

With the aging process, the structure and composition of the skin change as a result of environmental threats, such as ultraviolet exposure, air pollution and tobacco smoke, among others (extrinsic factors), as well as biological aging (intrinsic factors) [10]. Oxidative stress also contributes to both intrinsic and extrinsic skin aging [11]. Reactive oxygen species (ROS) produced as a result of ultraviolet exposure or metabolically generated increase the expression of metalloproteinases (MMPs) that degrade ECM proteins (ECMp) [12]. MMP levels are elevated in aged or photodamaged skin [13]. MMP-1 is the primary protease responsible for the fragmentation of type I and type III collagen, which are predominant in human skin [14], whereas MMP-12 is the most active in the degradation of elastin [15]. As a result of increased MMPs activity, collagen and elastin fibres are degraded and disorganised in aged skin. The increased degradation coupled with reduced biosynthesis results in a net collagen and elastin deficiency [16,17]. In terms of GAGs, deficiencies of HA and total sulphated GAGs have also been documented as a result of the aging process. Both intrinsic and extrinsic factors appear to downregulate HA synthases, resulting in a reduction of HA levels, particularly in the epidermis [18]. The reduction in the molecular weight (MW) of HA has been suggested to be mediated by ROS [19]. In parallel with HA, levels of total sulphated GAGs have been shown to be reduced in aged skin compared to young skin [20]. Thus, the aging process is associated with a reduction of epidermal and dermal thickness and quality [21], resulting in the appearance of fine wrinkles and flaccid, dry and dull skin (Figure 1).

Given the increasingly serious aging trend in today’s society, as well as the significant social burden and research investment, it is of great social and scientific significance to strengthen the development of anti-aging products. The most effective way to combat visible signs of skin aging and improve its appearance and quality is to provide effective ingredients that can reach the epidermis and dermis, both externally and internally, and inhibit the main mechanisms associated with aging [22,23].

HA is one of the most widely used biomolecules to treat skin aging. There are different sources of HA, such as microbial fermentation (F-HA) and extraction from mammalian and marine animals (E-HA). Both origins are the most standardized due to their high performance and optimization of the processes for obtaining them, during which the intrinsic properties of HA must be maintained. More recently, cell-free systems are being studied as a new source of HA, but these still present some process limitations and cannot yet be used for industrial production [24]. The process of obtaining HA mainly involves two phases: the extraction phase and the purification phase. The extraction phase of F-HA is carried out from the culture medium of bacterial strains (e.g., *Streptococci, Bacilli* and *Escherichia coli*) [25]. Then, the culture medium is ultrafiltered. Regarding the E-HA, it can be obtained mainly from tissues such as rooster comb, umbilical cord, vitreous humour and synovial liquid through a process of mechanical crushing and delipidation with ethanol [26,27]. Then, different procedures can be performed to continue with the extraction process of HA: the extraction method with hot water or with a diluted alkali–water solution (e.g., NaOH solution or NaCO_3_ solution) or through an enzymatic hydrolysis (using enzymes such as papain or alcalasa) [28]. Finally, F-HA and E-HA are purified by precipitation using organic solvents (acetic acid or ethanol), neutral salts (NaCl, KCl), metal ions (copper, calcium), quaternary ammonium salts of long chain (cetyl trimethyl ammonium bromide, cetylpyridinium chloride) or through the column chromatography method with a cellulose column, an anion exchange column or a gel column [24,29]. Based on the origin and the obtaining process of HA, its efficacy and safety can vary enormously [30,31]. This implies the need to evaluate the safety and efficacy of HA-based products in both in vitro and in vivo studies.

The aim of this study was to assess the anti-aging properties of an HA matrix ingredient containing a high concentration of HA, sulphated GAGs (specifically CS/DS) and collagen. To achieve this, we first extracted the HA matrix ingredient from rooster comb and its physicochemical characterisation was performed. Then, its regenerative, anti-aging and antioxidant properties were tested in vitro and finally, its intestinal absorption was evaluated.

## 2. Results

### 2.1. Extraction, Purification and Physicochemical Characterisation of the HA Matrix

The extraction process was performed on rooster combs through enzymatic digestion with Alcalase^®^, followed by filtration and concentration steps. Then, a purification process was performed by precipitation with NaCl, obtaining an off-white hygroscopic powder with a homogeneous appearance. Based on the dry weight, the extraction yield of the HA matrix was about 1%. The results of the physicochemical characterisation are summarised in Table 1.

### 2.2. Compositional Analysis of the HA Matrix

**Figure 2:**
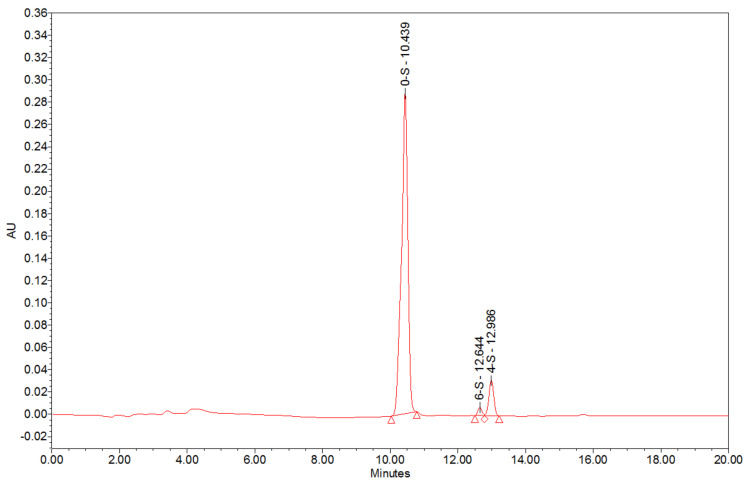
Chromatogram of analysis performed with SAX-HPLC with UV detection from HA matrix with Chondroitinase ABC digestion.

To analyse the composition of the purified HA matrix, a disaccharide analysis was first carried out. To identify the disaccharide profile, a specific treatment with chondroitinase ABC digestion was performed. The results showed that the HA matrix was composed mainly of non-sulphated disaccharides and minor amounts of 6-sulphated and 4-sulphated disaccharides (Figure 2).

Then, a quantitative analysis of the components present in the HA matrix (non-sulphated and sulphated GAGs) was performed. HA is the only non-sulphated GAG, and it is composed of glucuronic acid (always present) and acetyl-glucosamine. Thus, the HA content was calculated by quantifying the glucuronic acid of a sample by UV spectrophotometry. The results showed that the HA matrix content of the sample was 67%. In addition, the sulphated GAGs content was determined by SAX-HPLC and UV detection, which is specific to determine the sum of DS and CS. The results determined that the HA matrix sample contained 12% DS and CS. Both quantitative analyses confirm the disaccharide profile observed.

**Figure 3:**
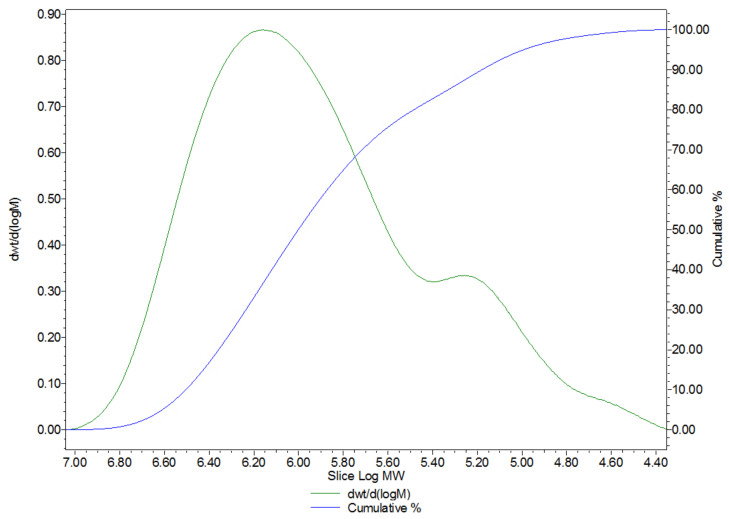
Molecular weight (MW) distribution of the HA matrix. In green, the abundance of fractions vs. the log MW. In blue, the cumulative fractions (percentage) vs. the log MW.

The characterisation of the MW of the HA matrix samples was performed by HPSEC with RI detection. The results showed three bands that correspond to HA, sulphated GAGs and proteins. The overall distribution had an average MW of about 1.3 MDa (Figure 3).

**Figure 4:**
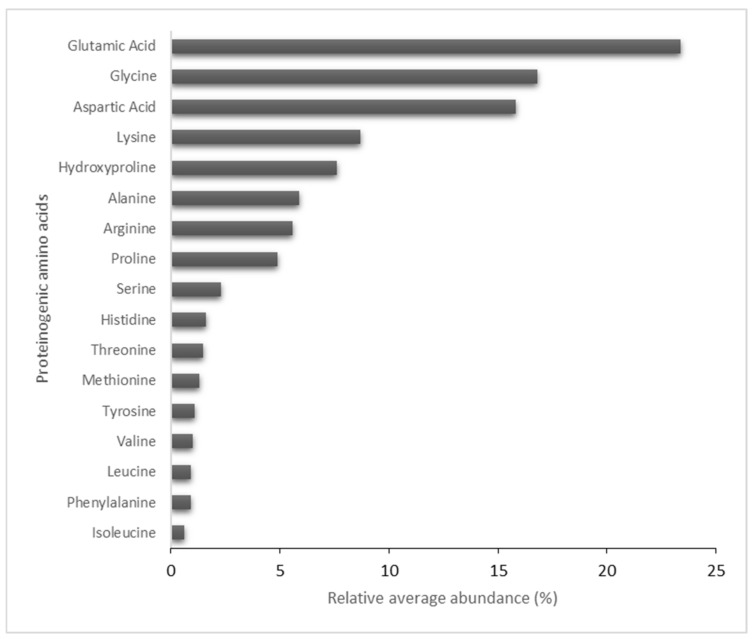
Relative average abundance of the amino acids profile of the HA matrix.

To determine the specific composition of the HA matrix, an evaluation and quantification of the amino acid content was performed. The total amino acid content of the sample was 17%. The total amount of the identified proteinogenic amino acids was considered 100% for calculations of relative abundance. As shown in Figure 4, the amino acid profile identified 17 proteinogenic amino acids in the HA matrix sample, the most relative abundance of which was glutamic acid/glutamine (23.4%), glycine (16.8%), aspartic acid/asparagine (15.8%), lysine (8.7%) and hydroxyproline (7.6%). The rest of the amino acids were found in lower, but still appreciable amounts (≤6%) (Figure 4).

In addition, the collagen content was evaluated from the hydroxyproline content, multiplying the hydroxyproline content (g/100 g) by 8, as established by the AOAC method 990.26 [29]. According to the results of the amino acid profile, the hydroxyproline content in the HA matrix was 1.3%, thus estimating a collagen content of 10.4%.

Compositional analysis of the HA matrix determined that it is mainly composed of HA, sulphated GAGs and protein.

### 2.3. HA Matrix Effects on Skin Cell Viability

To determine the appropriate concentrations required for the in vitro assays, a cytotoxicity test was performed to evaluate the effect of the HA matrix on HDF and HEK viability. The cytotoxic effects were evaluated through the MTT assay. The percentage cell viability was calculated with respect to NT, equivalent to 100%. As expected, the reference surfactant and cytotoxic agents, SDS, reduced the viability of HDF and HEK to about 10% at both tested times (24 h and 72 h). The decrease in cell viability both in HDF and HEK after treatment with the HA matrix did not exceed the pre-established value/threshold (20%).

**Figure 5:**
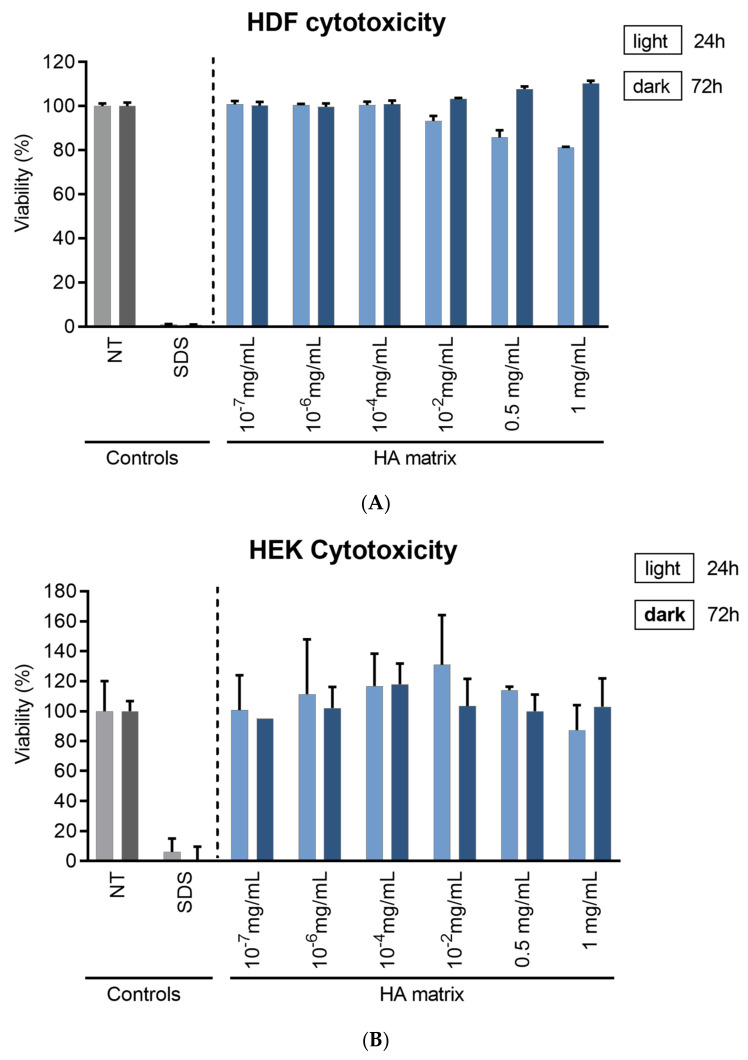
Cell viability of (**A**) HDF and (**B**) HEK after 24 and 72 h exposure to a range of HA matrix concentrations (10–7 mg/mL–1 mg/mL). The values correspond to the mean viability percentages (%) relative to the non-treated cells (NT).

Therefore, no impact on HDF or HEK viability was observed after 24 h and 72 h of exposure to the HA matrix (Figure 5). Nevertheless, there seems to be a trend of reduced HDF viability after 24 h and at the highest tested concentrations of the HA matrix. However, these differences in cell viability were not shown to be significant in comparison to those at 72 h. Likewise, the differences in HEK viability at the six different HA matrix concentrations tested were not significant. With the findings of the MTT assay, concentrations ranging from 0.05 to 1 mg/mL were selected for the in vitro studies, as these were the highest and non-cytotoxic concentrations tested of the HA matrix.

### 2.4. Stimulation of HDF and HEK Proliferation

**Figure 6:**
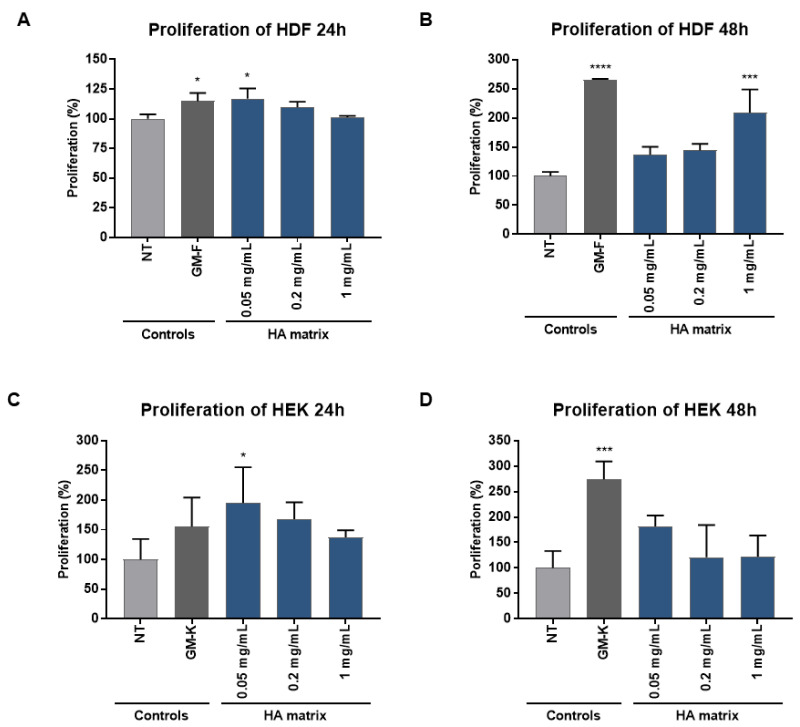
Effect of the HA matrix on HDF and HEK proliferation. Proliferation of (**A**,**B**) HDF and (**C**,**D**) HEK after 24 h and 48 h exposure to three HA matrix concentrations. The values correspond to the mean proliferation percentages (%) relative to the non-treated cells (NT). * *p* < 0.03; *** *p* < 0.0002; **** *p* < 0.0001.

The cell proliferation in HDF and HEK treated with the HA matrix was evaluated using Bromodeoxyuridine (BrdU) incorporation assay, which has long been used to detect in vitro DNA synthesis. The key principle of this method is that the BrdU incorporated as a thymidine analogue into nuclear DNA represents a label of the newly synthesised DNA strands in actively proliferating cells that can be tracked using antibody probes. The colorimetric reaction produced by this assay was detected by immunohistochemistry and was used for the determination of the HA matrix effect on skin cells proliferation. Increased proliferation of the HDF and HEK after HA matrix treatment compared to the NT was observed (Figure 6). The HA matrix (0.05 mg/mL) induced a significant increase (16.96 ± 8.58%) in HDF proliferation after 24 h of exposure. This increase was similar to the results of the HDF maintained in GM-F, showing an increment in their proliferation of 14.81 ± 6.84% compared to the NT. The HA matrix 1 mg/mL and GM-F remarkably induced HDF proliferation after 48 h of exposure, showing a 2.08 ± 0.40 and 2.66 ± 0.01-fold increase compared to the NT, respectively. The lowest HA matrix concentrations tested (0.05 and 0.2 mg/mL) did not show significant effects on HDF proliferation at this exposure time (48 h).

### 2.5. Effects of HA Matrix on Cell Migration

Regarding HEK proliferation, the HA matrix 0.05 mg/mL significantly induced an increment of 108.58 ± 40.36% after 24 h of exposure, whereas GM-K did not show any significant effect. Conversely, the HA matrix did not exert any significant effect at 48 h of exposure, whereas a 2.74 ± 0.35-fold increase was observed after exposure to GM-K. It should be noted that the increase in HEK proliferation induced by 0.05 mg/mL HA matrix was similar at both exposure times evaluated (24 h and 48 h). However, based on the statistical analysis performed and considering the values obtained, especially for the GM-K control, the HA matrix-related increase observed at 48 h was not identified as statistically significant.

**Figure 7:**
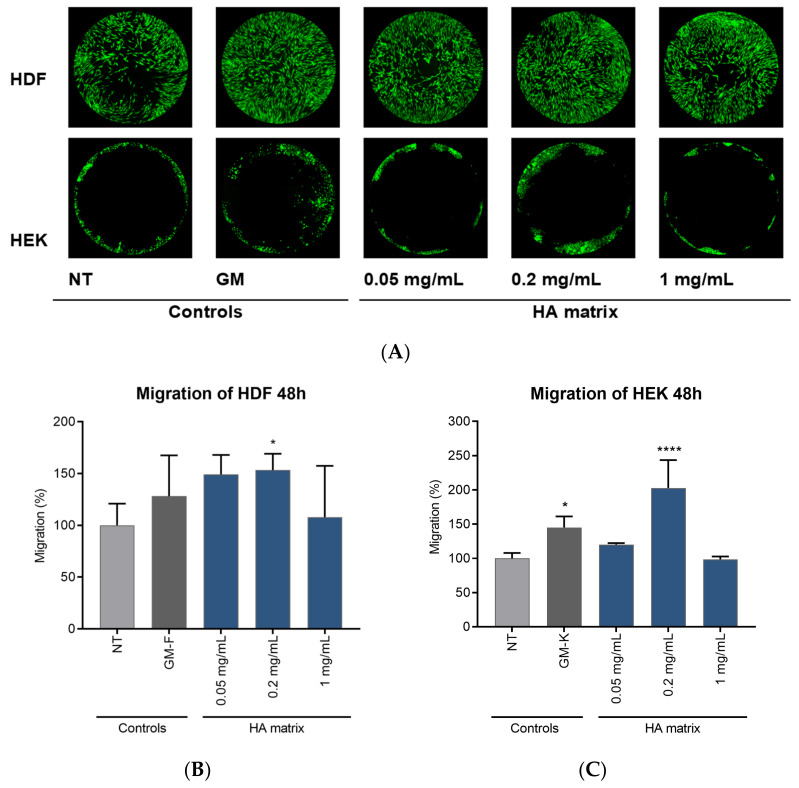
Effects on HDF and HEK migration after 48 h of exposure to three HA matrix concentrations. (**A**) Images of cell migration after 48 h of exposure to the product. Mean percentage (**B**) HDF and (**C**) HEK migration with respect to the non-treated cells (NT). * *p* < 0.03; **** p < 0.0001.

The cell migratory capacity of the HA matrix was determined through fluorescence quantification of calcein-labelled cells migrated to the free-seeded area of the well in the Oris™ 96-well migration plate. The influence of HA on HDF and HEK migration is shown in Figure 7, in which the effect of GM is also depicted. The GM-F did not exert any significant effect on HDF migration, whereas the GM-K stimulated HEK migration, increasing their migration rate by 45.20 ± 15.99% compared to the NT cells. The treatment of HDF with the 0.2 mg/mL HA matrix concentration showed a significant induction of migration, with an increase of 49.22 ± 18.83%. Although a marked increase in HDF migration was also observed after the 0.05 mg/mL HA matrix, this was not statistically significant. A major deviation was observed at the highest concentration tested, 1 mg/mL.

Regarding HEK, a significative and remarkable 2-fold increase of the cell migration rate was observed after 48 h of exposure to the HA matrix 0.2 mg/mL compared to the NT. The HEK exposed to the lowest tested HA concentration (0.05 mg/mL) did not show differences with respect to the NT cells. Likewise, the highest tested concentration of the HA matrix (1 mg/mL) did not exert an effect on HEK migration, similar to the results observed in the cell proliferation study. Based on these results, it is possible that, despite the absence of a cytotoxic effect, the HEK were sensitive to the concentration of 1 mg/mL of HA matrix, which limits the observation of effects on this cellular mechanism.

### 2.6. Induction of ECMp Production

**Figure 8:**
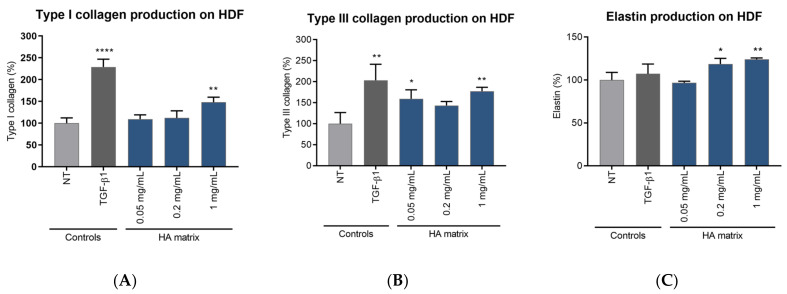
HA matrix stimulation of ECMp production on HDF. Mean percentage production of (**A**) type I Collagen, (**B**) type III Collagen and (**C**) elastin with respect to the non-treated cells (NT). * *p* < 0.03; ** *p* < 0.002; **** *p* < 0.0001.

The effect of the HA matrix on ECMp production was evaluated through indirect ELISA quantification on HDF, evaluating the levels of mature type I and type III collagen and elastin as functional components of the ECM of the cultured HDF. The HA matrix showed a significantly stimulatory effect of ECMp production on HDF, increasing the synthesis of the three evaluated proteins after 72 h of treatment compared to the NT (Figure 8). Specifically, HDF treated with the highest tested HA matrix concentration (1 mg/mL) showed a significant increase of 48.04 ± 11.49% of type I collagen. Type III collagen production in HDF was significantly increased with 0.05 mg/mL and 1 mg/mL of HA matrix, 59.28 ± 21.31% and 77.27 ± 9.28%, respectively, compared to the NT. Finally, the quantification of elastin on HDF showed a significant increase of 18.53 ± 6.70% and 24.09 ± 1.57% after treatment with 0.2 mg/mL and 1 mg/mL, respectively.

TGF-β1, a gold standard growth factor in the induction of ECMp synthesis, did not exert any significant effect on elastin production after 72 h. In contrast, the HA matrix was able to induce the production of this protein under the same experimental conditions. Conversely, the HDF treated with TGF-β1 showed a 2.28 ± 0.18 and 2.03 ± 038-fold increase in type I and type III collagen production, respectively, compared to the NT cells. Although this stimulatory effect of TGF-b1 was slightly higher than that of the HA matrix, it should be noted that this is a growth factor with a powerful and directed action on the synthesis of these two ECMp.

### 2.7. Induction of Glycosaminoglycan Synthesis

**Figure 9:**
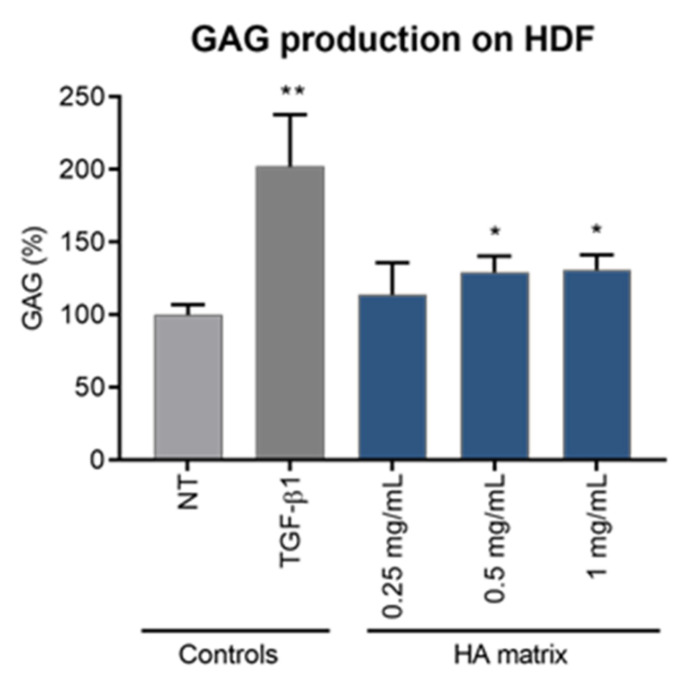
Effects of the HA matrix on GAGs production on HDF after 24 h. Mean percentage values of GAGs production with respect to the non-treated cells (NT). * *p* < 0.03; ** *p* < 0.002.

The ability of the HA matrix to modulate the synthesis of GAGs was studied on HDF by radiolabelling technique. The cells were treated with three concentrations (0.25, 0.5 and 1 mg/mL) of the HA matrix and newly synthesised GAG molecules were assessed by measuring the incorporation of [3H] glucosamine after 24 h of exposure (Figure 9). TGF-β1 was included as a reference molecule with a stimulatory activity on GAG synthesis. The results showed a significative increase of de novo GAG synthesis on HDF after exposure to the different treatments. As was expected, TGF-β1 stimulated GAG synthesis, as HDF treated with this growth factor showed a 2.02 ± 0.36-fold increase in GAG levels compared to the NT. The HA matrix stimulated GAG synthesis at the highest tested concentrations (0.5 mg/mL and 1 mg/mL), with GAG levels being 29.57 ± 10.92% and 30.89 ± 10.52%, respectively, higher in the product-treated HDF than in the NT.

### 2.8. Antioxidant Activity

**Figure 10:**
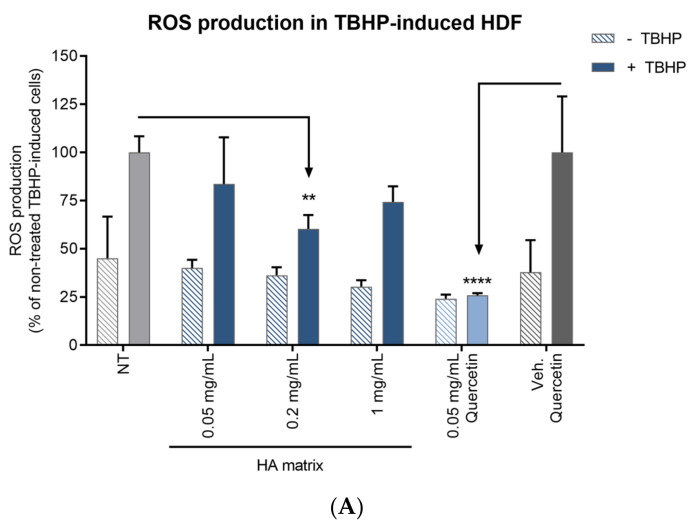

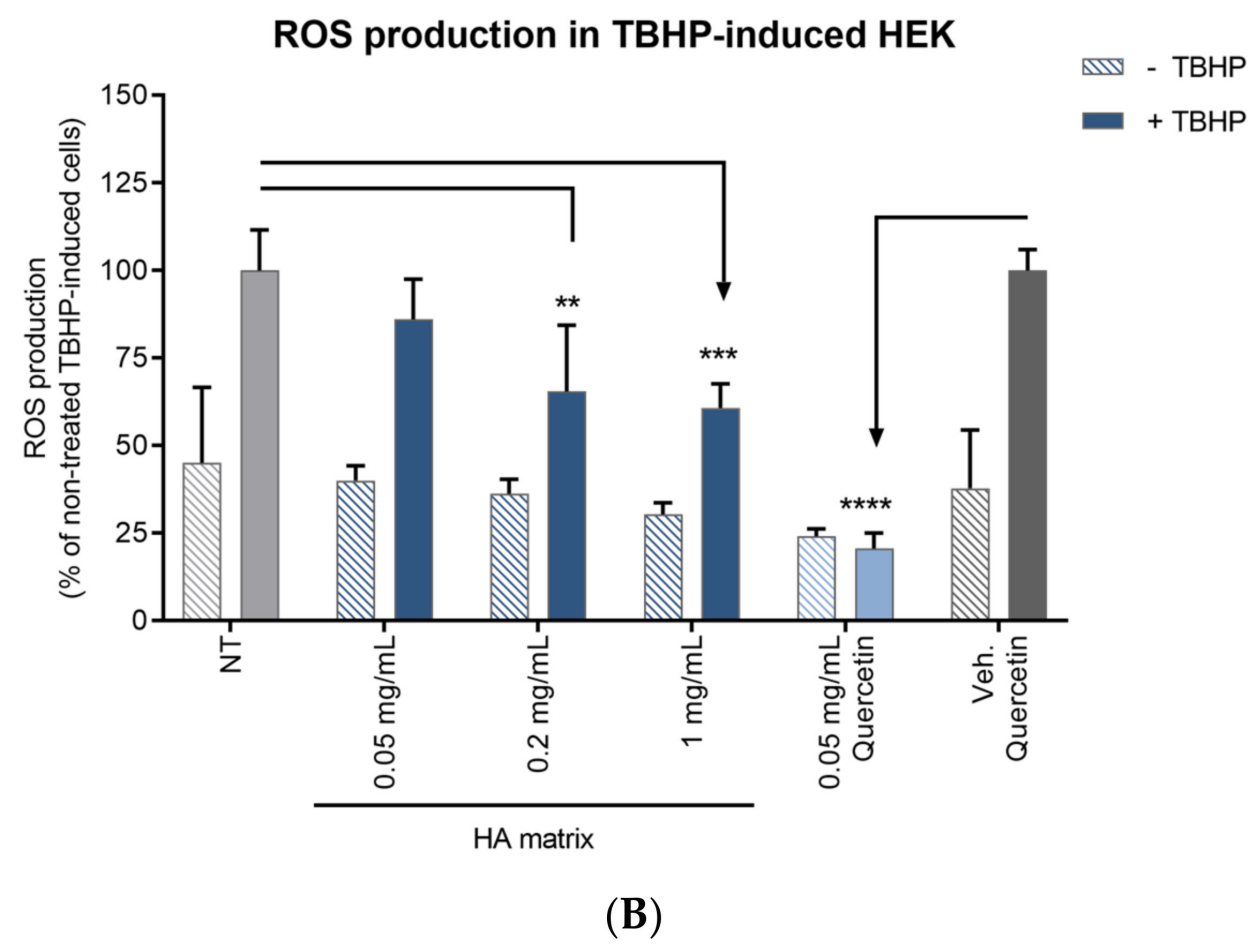
Effects of the HA matrix (0.05 mg/mL, 0.2 mg/mL and 1 mg/mL) on the production of ROS on (**A**) HDF and (**B**) HEK 4 h after treatment. Mean percentage ROS production compared to the non-treated (+/−TBHP) cells. ** *p* < 0.002; *** *p* < 0.0002; **** *p* < 0.0001.

The antioxidant activity of the HA matrix was assessed by measuring intracellular ROS production on HDF and HEK. The ROS levels were determined by measuring cell staining with Carboxy-H_2_DCFDA in the absence or presence of an oxidative stimuli (TBHP). Both the TBHP-induced HDF and HEK showed a 2-fold increase in ROS production compared to non-TBHP-induced cells, proving the proper oxidative stress response was induced by TBHP in both models (Figure 10).

The ROS production in the HDF treated with 0.2 mg/mL HA matrix significantly decreased (39.73 ± 7.23%) compared to the NT HDF after oxidant induction with TBHP. On the other hand, the decrease of intracellular ROS production in HEK showed a significant linear trend, in a concentration dependent manner, in the presence of the HA matrix. ROS production significantly decreased by 34.58 ± 18.97% and 39.27 ± 6.94% at 0.2 and 1 mg/mL of HA matrix, respectively, compared to the NT TBHP-induced HEK. As the ROS levels in non-TBHP-induced cells showed, the HA matrix did not show any pro-oxidant effect over either of the two cellular models under the tested conditions. Quercetin showed a similar effect on both cell types, reducing the ROS levels to 25.86 ± 1.27% in HDF and 20.65 ± 4.36% in stressed HEK compared to the NT TBHP-induced cells.

### 2.9. In Vitro Absorption of the HA Matrix

The absorption was assessed by measuring the GAG concentration inside the everted-gut sac rat model, which were incubated in a physiological medium containing the HA matrix for 5, 10, 20 or 30 min.

**Figure 11:**
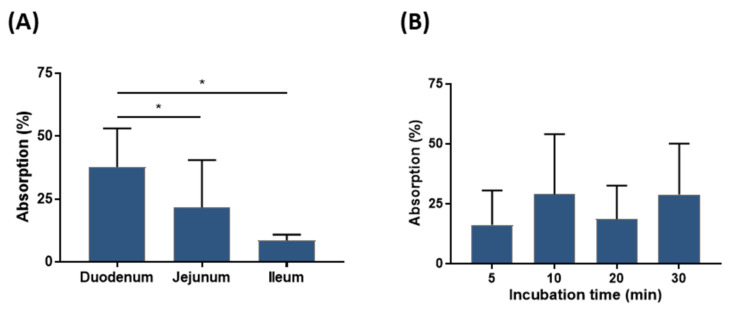
Mean ± SD GAG concentration, and the corresponding degree of absorption (%) in each intestinal region (**A**), and at each incubation time (**B**) after incubation in KB medium containing 200 µg/mL HA matrix. * *p* < 0.05.

The degree of absorption (percentage of internal concentration relative to the nominal concentration of the incubation medium) in each sac was calculated. The results are depicted in Figure 11 as the % absorption ± SD in each region and at each incubation time. The coefficients of variation (CV) between GAG concentrations were high, both for the data analysed by incubation time (around 80%) and for the data analysed by intestine region (between 27% and 87%). The data analysis by incubation time did not show significant differences, although apparent differences in the values can be seen. On the other hand, the analysis by each intestine region suggests that there was GAG absorption in the three regions of the intestine tested: 38 ± 15% absorption in the duodenum, 22 ± 19% in the jejunum and 9 ± 2% in the ileum. These results showed the absorption differences between the ileum and duodenum, and between the jejunum and duodenum, while no differences between the ileum and jejunum were detected.

## 3. Discussion

With aging, human skin undergoes a physiological decline that affects its mechanical, protective and restorative abilities. At the dermal level, the synthesis of new ECM components by dermal fibroblast slows down, resulting in an inadequate replacement of the degraded ECM. Aged dermal fibroblasts also lose proteostasis, which leads to the production of an aberrant ECM [32]. The dermal collagen network becomes increasingly fragmented, with shorter and less organised fibres and an increased expression of MMPs, which are responsible for collagen degradation [33]. Elastin fibres become degraded and lose elasticity, leading to a loss of the skin’s structural integrity [34]. Another major component of the dermal ECM, and also present in the epidermis, is the group of GAGs, such as HA, CS and DS, which decrease with age [20] and reduce the skin’s ability to retain moisture. At the epidermal level, the most noticeable effect of aging is the thinning and impairment of the epidermal barrier. This leads to a decrease in collagen density, loss of elasticity in elastin fibres and a lower amount of HA and sulphated GAGs, such as CS and DS, resulting in dry skin and a comprised epidermal barrier function. The manifestation of skin aging includes the appearance of wrinkles, spots, dryness, roughness and a reduced barrier integrity, as well as thinning of the epidermis [35].

The formulation of cosmetics and nutricosmetics with HA represents one of the main anti-aging treatments. Rooster comb is one of the most common sources of pure HA, obtained after a lengthy purification process [36]. For this study, the process of extraction and purification from the comb of a rooster was used to obtain not only HA, but also GAGs and collagen, thus generating an ingredient matrix composed of three biomolecules with anti-aging properties [24]. In fact, a recent study has demonstrated the superior effect of this specific ingredient as compared to pure HA from extraction or fermentation origin [37].

From a physicochemical perspective, the HA matrix extracted from the rooster comb had optimal physiochemical characteristics. The particle size of the powder was confirmed to be 100% below 600 µm and the pH and moisture levels were within the expected range for purified sodium hyaluronate [38]. The levels of salts present in the samples were less than 1% for chloride. The nitrogen levels can be attributed to the presence of HA, the protein content (amino acids) and the sulphated GAGs in the sample.

Compositional analyses of the sample revealed that the HA matrix isolated and purified from the rooster comb is composed of HA (>60%); sulphated GAGs (10%), including DS and CS; protein (17%), including collagen (10.4%); and water. These components are naturally present in rooster combs as proteoglycans in the cartilage. Enzymatic digestion with protease releases HA and sulphated GAGs from the proteoglycans by hydrolysing protein, and subsequent steps of the process isolate the fraction rich in HA, with minor constituents, such as sulphated GAGs and hydrolysed protein, mainly composed of collagen. All the components identified in the HA matrix are functional components in the skin’s extracellular matrix. On the one hand, HA and sulphated GAGs have the capacity to absorb up to 1000 times their volume in water, helping to retain moisture and prevent water loss at both the epidermal and dermal levels [6,39]. On the other hand, collagen provides the dermal layer with tensile strength and elasticity [6].

HA is the major component of the characterised matrix and plays a variety of biological roles, including anti-inflammatory, wound healing, tissue regeneration and immunomodulation [40,41,42]. Many of these biological properties of HA are thought to be related to its molecular characteristics, particularly its MW and the way it is synthesised or degraded [43,44]. The average MW of the HA matrix was 1.3 MDa, which can be considered a high molecular weight HA (HMW-HA, >1 million Da). HMW-HA has anti-inflammatory and immunosuppressive properties [42,45].

In vitro studies were conducted to evaluate the regenerative, anti-aging and antioxidant activity of the HA matrix. The results show that the HA matrix stimulates the migration and proliferation of HDF and HEK. These cellular properties improve the rate of cell replenishment, providing a regenerating effect on the epidermis and dermis. These findings are in line with previous reports [40,46,47,48,49] that highlight the stimulative properties of GAGs and collagen on cell migration and proliferation. Additionally, prior research suggests the modulative effect of collagen and collagen-derived compounds on the skin’s ECM. Zague et al. [50] investigated the influence of collagen hydrolysate on the ECM metabolism of HDF derived from chronological aged and photoaged skin. The reported results show that collagen hydrolysate, derived from bovine type I collagen, notably modulated cell metabolism, increasing the content of type I procollagen and collagen by stimulating their biosynthesis and inhibiting MMP-1 and MMP-2 on both chronological aged and photoaged HDF. In Hwang et al.’s [51,52] studies, human alpha-1 and alpha-2 type I collagen (COL1A1 and COL1A2)-derived peptides revealed stimulatory effects on type I collagen and elastin production on HDF. Both COL1A1 and COL1A2-derived peptides exerted the same effect promoting collagen synthesis and elastin production. The levels of secreted type I collagen and elastin were significantly increased after treatment with both COL1A1 and COL1A2-derived proteins and COL1A2-derived peptides. Consistent with these results, the HA matrix showed a positive effect inducing type I collagen, type III collagen and elastin synthesis on HDF. However, it must be pointed out that, whereas most of the studies from the literature quantified the released proteins or precursors of ECMp, in this study, the quantification is performed on the fixed HDF cell monolayer, where the quantified proteins are localised and integrated into the ECM in their mature and functional form. ECMp, especially the structural proteins type I and type III collagen, along with elastin, play a critical role in maintaining skin firmness and elasticity, since the synthesis and stability of these proteins decline with age.

Regarding the production of GAGs, Ohara et al. [49] reported the stimulative effect of a collagen-derived peptide, Pro-Hyp, derived from porcine type I collagen, over HAS2 mRNA expression and hyaluronan production in HDF. In the present study, the HA matrix also stimulated GAGs synthesis on HDF. Since GAGs are hydrophilic complexes with hygroscopic properties [53], the reported results might be an initial indication of the skin moisturising effects of the HA matrix. The stimulative activity of the presented multicomponent matrix on the production of collagen, elastin and GAGs may help maintain a stable collagen ratio, repair the skin structure and maintain the skin’s moisture content, preventing and treating aging signals and enhancing a youthful appearance.

Oxidative stress is another key event associated with skin aging, often due to an excessive increase in ROS. ROS play a significant role in skin aging [11], as their accumulation causes DNA damage, induces the skin’s inflammatory response, reduces antioxidant enzymes, indirectly inhibits collagen production and increases MMPs, leading to the degradation of ECM elements in the dermis (collagen, binding proteins, sulphated GAGs and HA) [11,19,34,54].

The HA matrix showed an antioxidant effect on HDF and HEK, which was more pronounced in HEK. Several studies have related ROS levels to ECM degradation [11,55]. Hence, the antioxidant potential of the HA matrix has been evaluated. HA, mainly HMW-HA, has shown antioxidant activity reducing ROS levels in a wide range of cell types, including nasal conjunctival cells, hepatic cells, white blood cells [56], chondrocytes [57] or human immortalised keratinocytes (HaCaT). At the epithelium level, Albano et al. [58] evaluated the antioxidant effect of LMW-HA (500 kDa), MMW-HA (~900 kDa) and HMW-HA (~1600 kDa) in an in vitro model of oxidative stress with nasal epithelial cells; ROS production and NADPH oxidase 4 (NOX-4) expression were determined. Pre-treatment with HMW-HA and MMW-HA reduced the induction of ROS production in IL-17A-stimulated cells compared to the cells treated with rhIL-17 alone, with the reduction of ROS levels being more pronounced in the HMW-HA pre-treated cells. Conversely, pre-treatment with LMW-HA did not exert any effect on the ROS levels in the cells treated with rhIL-17 alone. Pre-treatment with HMW-HA significantly inhibited NOX-4 synthesis, whereas MMW-HA and LMW-HA did not control the activity of rhIL-17A on NOX-4 synthesis. Albano et al. suggested that HMW-HA control oxidative stress by blocking ERK1/2 intracellular signal activation involved in the NF-ĸB transcriptional mechanism regulation. In another study performed by Ye et al. [59], HA was shown to be a protective agent against thimerosal (Thi), demonstrating antioxidant properties on Thi-induced conjunctival cells. Cells pre-treated with HA showed significantly decreased ROS production compared to cells exposed to Thi alone. Avadhani et al. [60] compared the protective effect of different nano-transfersomal formulations on UV-induced ROS activity in HaCaT cells. ROS generation was significantly reduced after treatment with nano-transfersomal formulations of epigallocatechin-3-gallate (EGCG) and EGCG with HA than UV irradiated cells. Interestingly, the reduction in ROS was more pronounced in the transfersomes containing HA. For the three studies, intracellular ROS levels were determined by measuring the oxidative conversion of cell-permeable carboxy-H2DCFDA to fluorescent DCF. In the present study, the antioxidant effect of the HA matrix was evaluated in both TBHP-induced HDF and HEK. The multicomponent matrix was able to lower the ROS levels in both the TBHP-induced HDF and HEK models, providing data on its protective effect against oxidative stress. The presented HA matrix showed a distinctive effect compared to the other HA discussed, since it was capable of controlling the ROS levels without the need for pre-treatment, showing a direct antioxidant action at the intracellular level. Moreover, even though previous in vitro studies have reported the antioxidant activity of HA in a wide range of cell line types, including keratinocytes (HaCaT) [60], this is the first study showing its activity in HDF and HEK.

The in vitro studies performed on HDF and HEK showed that the novel HA matrix (composed of HA as well as GAGs and collagen) induced cell proliferation and migration, increased the synthesis of collagen I and III, elastin and GAGs and had a protective effect against oxidative stress. These results suggest the anti-aging properties of the HA matrix due to its action on cellular and molecular mechanisms that are altered in aged skin. Likewise, these in vitro regenerative, anti-aging and antioxidant properties of the new complex HA matrix indicate its potential to improve skin conditions, such as hydration, elasticity, firmness and dermal density, although more in-depth studies and, especially, clinical tests would be necessary to confirm this.

There is growing interest in oral supplementation to correct HA deficiency in aged skin and, consequently, improve skin properties and appearance. Although oral administration is the most convenient route, absorption and bioavailability have been questioned. Previous studies have demonstrated absorption of HMW-HA [61], but there is some controversy about the effect of MW on the rate of absorption [62], as well as the potential contribution of intestinal fermentation [63]. Nonetheless, different human intervention studies have demonstrated certain anti-aging effects at the skin level as a result of oral HA supplementation [5,64,65,66,67]. In addition, taking into account that, besides HA, sulphated GAGs and collagen deficiencies have also been described in the skin as a result of the aging process, the intestinal absorption of an HA matrix was evaluated in order to explore its oral administration. This study was performed through an ex vivo everted-gut sac assay, which was used to test the absorption behaviour of drugs and nutrients [68]. The rates of transfer of the HA matrix throughout different regions of intestinal mucosa (duodenum, jejunum and ileum) at different incubation periods were performed to evaluate the degree of absorption of GAGs, which are the main components of the HA matrix. The GAG concentrations in the content of each incubated sac were analysed to calculate the percentage absorption by comparison with the nominal concentration of GAG in the incubation medium. High variability in GAG concentrations was obtained even between sacs from the same region at the same incubation time due to the limitations of this model (such as: method of euthanasia, harvesting time, intestinal region and experimental factors such as pH, aeration and temperature [68]).

Despite the limitations, the degree of transfer or absorption of the HA matrix was analysed in the three regions of the small intestine. Quantifiable GAG concentrations in most samples suggest a certain degree of absorption of GAGs from the medium in all sections of the intestine. The results show that absorption values decrease along the length of the small intestine. In fact, transfer in the jejunum and ileum (22% and 9%, respectively) showed significant differences compared to the duodenum (38%), but no significant differences between them. Although the highest transfer of substances is probably expected in the jejunum, as it has the largest mucosal surface area [69], some significant nutrients, such as calcium, iron and vitamin D, have been known to be transferred through the duodenum [70,71]. No evolution of concentrations was observed over time, as no significant differences were detected between incubation times, probably due to high variability.

The results obtained in the present study suggest that there could be absorption of the HA matrix in the intestine, which is consistent with that stated in the literature about GAGs oral absorption and disposition. Although the absorption of oral HA seems to be controversial [63,72], some recent studies suggest that HA matrix components (HA, CS, DS and Col) are absorbed in the intestine, reaching not only the bloodstream, but also the lymph, in the case of HA [62,72,73], and that they are subsequently distributed throughout the tissues, including the skin [5,63,74]. Due to the limitations of the model, further studies are needed to corroborate this oral absorption.

Unlike other HAs, the HA matrix developed has shown positive effects on collagen type I and type III and elastin synthesis. Its intestinal absorption has also been evaluated. On the other hand, it should be noted that one of the main differences of this study compared to others is that the in vitro tests were carried out in two cell types, keratinocytes and fibroblasts. The next logical step would be to conduct a clinical trial.

## 4. Materials and Methods

### 4.1. Extraction and Purification of HA Matrix

The HA matrix ingredient (Dermial^®^, Bioiberica S.A.U., Barcelona, Spain) was obtained from rooster combs sourced from poultry declared fit for human consumption in authorised slaughterhouses. The combs were cleaned of any adjacent bone or other tissues, such as skin of the head, fat or feathers. Then, they were washed with water and mechanically crushed. The manufacturing process involved an enzymatic hydrolysis of the rooster combs with a proteolytic enzyme, such as Alcalase^®^, from a non-genetically modified strain of Bacillus licheniformis (Sigma-Aldrich, St Louis, MO, USA). The enzyme was added in successive stages until all the material was digested under agitation conditions. After the hydrolysis step, the enzyme was inactivated by heat-treatment. Once hydrolysation was completed, the digested combs underwent a heat treatment step at a minimum temperature of 82 °C, which ensured the microbiological safety of the product. The resulting product was then filtered and concentrated using a vacuum. Finally, the product was precipitated with NaCl (discarding the supernatant), dried and milled [24].

### 4.2. Physicochemical Characterisation

#### 4.2.1. Macroscopic Characteristics

The appearance of the product was assessed by visually inspecting the sample to describe its colour and texture.

#### 4.2.2. Granulometry

The particle size was determined on a 10-g sample using an analytical sieve (600 µm) and the results are reported as the percentage of material that passed though the sieve [75].

#### 4.2.3. pH Determination

The pH was measured with 0.5% *w/v* solution of the product in purified water (Merck-Millipore, Darmstadt, Germany) using a pH meter with a combined-glass electrode (Metrohm, Herisau, Switzerland) [38].

#### 4.2.4. Chloride Levels Determination

The chloride levels were determined in 67 mg of the product in 100 mL of water by precipitation using silver nitrate (Panreac, Barcelona, Spain), and assessing the turbidity formed against a known concentration of chloride ion [38].

#### 4.2.5. Nitrogen Content

The nitrogen content was determined using the Kjeldahl method [76]: 0.5 g of the sample was mineralised with sulphuric acid (Panreac, Barcelona, Spain) using a copper sulphate–selenium catalyst (Merck, Darmstadt, Germany). The ammonium ion was distilled by adding 40% NaOH solution (Carl-Roth, Karlsruhe, Germany) with air flow, solubilised in 4% boric acid solution (Quimivita, Barcelona, Spain) and titrated with 0.1 N sulfuric acid (Panreac).

#### 4.2.6. Loss on Drying

Loss on drying was determined by drying a 0.5 g sample at 60 °C for 6 h under a vacuum using phosphorus pentoxide as a desiccant [77].

### 4.3. Compositional Analysis of Hyaluronic Acid Matrix Ingredient

#### 4.3.1. Disaccharide Profile

The disaccharide profile was analysed by SAX-HPLC with UV detection [78]: 250 mg of the sample diluted to 100 mL with water was digested with Chondroitinase ABC (Sigma-Aldrich, St Louis, MO, USA) solutions in TRIS buffer at 37 °C for 3 h. Then, 25 µL of the digested solutions were injected into a Waters HPLC System (Waters, Milford, MA, USA), consisting of a Waters Alliance 2695 with a Waters 2489 variable wavelength detector set at 230 nm. The column used was Waters Spherisorb 5 μm SAX (Waters). The gradient was from water to 0.39 M sodium chloride at 1 mL/min and the pH of the mobile phases was adjusted to pH 3 with hydrochloric acid. Empower 3 (Waters) software was used for data acquisition and reporting.

#### 4.3.2. HA Content

The HA content was calculated from the glucuronic acid content [38]. The glucuronic acid content was determined by reacting glucuronic acid with carbazole [38,79] based on the conditions published by Bitter and Muir [79]. Then, 1 mL of the sample solution (0.2 mg/mL in water) was hydrolysed with 5 mL of a solution containing 38.16 g of sodium tetraborate (Merck) in 500 mL of sulfuric acid (Panreac) at 95–100 °C for 30 min. The solution was cooled in an ice bath and then 0.20 mL of a solution of carbazole (Sigma-Aldrich) of 1.25 µg/mL in absolute ethanol (Panreac) was added, mixed by vigorously shaking and heated at 90–95 °C for 30 min. After this, the solution was cooled to room temperature, and the absorbances were read at 520 nm using a 630 UV spectrophotometer (Jasco, Hachioji, Tokyo, Japan). The content was calculated using a calibration curve generated with the glucuronic acid (Sigma-Aldrich) solutions with concentrations ranging from to 10 to 100 µg/mL. Water was used as the blank. Both the standard and blank solutions followed the same process as the sample solution.

#### 4.3.3. Sulphated GAGs Content

The sulphated GAGs were determined by SAX-HPLC with UV detection [80]: 125 mg of the sample was diluted to 25 mL with water and analysed using an IonPak AS-11 column with an AG-11 guard column (Waltham, MA, USA). The mobile phase was prepared with sodium dihydrogen phosphate (Panreac) at pH 3. The gradient at 0.22 mL/min was performed with sodium perchlorate (Panreac) adjusted to pH 3. The DS and over-sulphated CS were used as the standards (EDQM, Strasbourg, France), previously diluted to 1 mg/mL. The 20 µL standard solutions and samples were injected into an Alliance 2695 HPLC system (Waters) equipped with a Waters 2489 variable wavelength detector (Waters) set at 202 nm and a column oven set at 40 °C. Empower 3 software (Waters) was used for data acquisition and reporting.

#### 4.3.4. Molecular Weight Determination

The MW of the HA was determined by HPSEC with RI detection. The sample solution 1 mg/mL in the mobile phase was injected into an Alliance 2695 HPLC system (Waters) equipped with a Waters 2414 RI detector (Waters). The column was a TSK Gel 6000 PWXL (Tosoh, Tokyo, Japan), the mobile phase was 0.1 M sodium chloride (Merck) at 0.4 mL/min and the column and detector temperature was set at 37 °C. Empower 3 software (FR5, DB version 7.50) with a GPC module (Waters) was used for data acquisition, calculation and reporting.

#### 4.3.5. Aminogram

The amino acid content was determined by HPLC (Waters) with fluorescence detection using precolumn derivatisation with 10 g of the product. The steps consisted of acidic hydrolysis in hydrochloric acid with 1% phenol (Panreac) at 100 °C for 24 h. Derivatisation of primary amino acids was performed with OPA/2-mercaptoethanol (Merck). Secondary amino acids were derivatised with 9-Fluorenylmethoxycarbonyl chloride (FMOC-Cl, Sigma-Aldrich) and HPLC separation was performed in a C18 column (20 cm length, 5 µm) [81].

### 4.4. HA Matrix Biological Activity Assessment

#### 4.4.1. Cell Cultures

In vitro studies were performed on primary human dermal fibroblasts (HDF; Lonza, Basel, Switzerland) and primary human epidermal keratinocytes (HEK; Cascade Biologics, Portland, OR, USA). Both cell types were isolated from foreskin samples, surpluses from surgery of neonatal donors.

The cells were cultured in their respective growth medium (GM). The HDF were cultured in growth medium (GM-F) consisting of Dulbecco’s 1 g/L glucose medium (DMEM; Sigma-Aldrich) supplemented with 10% foetal bovine serum (FBS; PAA Laboratories, Westborough, MA, USA), 2 mM L-glutamine (Lonza) and 100 μg/mL penicillin—100 U/mL streptomycin (Lonza). The HEK were cultured in growth medium (GM-K) composed of Epilife^®^ Medium (Thermo Fisher Waltham, Scientific, MA, USA) with calcium chloride (0.06 mM) and supplemented with the Human Keratinocyte Growth Supplement kit (Thermo Fisher Scientific): bovine pituitary extract (0.2% *v*/*v*), recombinant insulin-like growth factor type 1 (rIGF-1, 1 μg/mL), hydrocortisone (0.18 μg/mL), bovine transferrin (5 μg/mL), epidermal growth factor (EGF, 0.2 ng/mL) and Gentamicin (0.2% *v*/*v*).

The HDF and HEK were routinely subcultured by washing twice with sterile phosphate buffered saline (PBS pH 7.4; Sigma-Aldrich), harvesting with trypsin–EDTA (0.025% trypsin and 0.01% EDTA; Thermo Fisher Scientific) and manually counting in a Neubauer chamber before seeding in a new cell culture flask (75 cm^2^, Falcon). Both cell lines were maintained at 37 °C in a humidified atmosphere containing 5% CO_2_ (standard culture conditions, SCC). For in vitro assays, HDF and HEK were seeded on cell culture plates. Once a correct density was reached, the cells were maintained on deprivation overnight. The serum-deprived medium consisted of DMEM supplemented with 0.1 FBS, 2 mM L-glutamine and 100 μg/mL penicillin—100 U/mL streptomycin for HDF and GM-K without rIGF-1 and EGF for HEK.

The analysed compounds were freshly prepared for each assay in the assay medium (AM) or Hanks’ Balanced Salt Solution (HBSS, Sigma-Aldrich) for ROS assay. The AM for HDF (AM-F) consisted of DMEM with 0.1 to 1% FBS, 2 mM L-glutamine and antibiotics (100 μg/mL penicillin and 100 U/mL streptomycin). The AM-F was supplemented with 0.1% FBS for the cytotoxicity and cell proliferation assays, while for the cell migration and ECMp and GAG production studies, it was supplemented with 1% FBS. The AM for HEK (AM-K) was GM-K without rIGF-1 and EGF.

#### 4.4.2. In Vitro Cytotoxicity Assay

The cytotoxicity of the HA matrix ingredient was assessed prior to conducting the bioactivity studies. The cytotoxicity was evaluated using the 3-[4,5-dimethylthiazol-2-yl-2,5-diphenyltetrazolium bromide (MTT, Sigma-Aldrich) colorimetric assay. HDF and HEK were seeded in 96-well culture plates at a final density of 5500 and 8000 cells/well, respectively. Then the cells were maintained in their specific GM until reaching 80% confluence. The cells were deprived and exposed to six different concentrations of HA matrix (10^−7^, 10^−6^, 10^−4^, 10^−2^, 0.5, 1 mg/mL in AM ng/mL prepared in AM-F with 0.1% FBS) for 24 h and 72 h. In parallel, non-treated cells (NT) were maintained in their respective AM and tested as the negative control, and the cells exposed to the cytotoxic surfactant, sodium dodecyl sulphate (SDS; 0.02%, Sigma-Aldrich), were used as the positive control. Then, the test solutions were removed, and the cells were incubated with 100 µL/well of the MTT solution (MTT 0.05 mg/mL in AM) for 2 h for HDF and 3 h for HEK in SCC and protected from light. The absorbance (540 nm) was measured with the Safire2 multi-detection plate reader (Tecan, ZU, Switzerland). The cell viability was calculated by measuring the differences in relative fluorescence units (RFU) of the tested product with respect to the NT cells. Treatments that reduced viability by more than 80% were considered cytotoxic [82,83,84].

#### 4.4.3. Cell Proliferation

Cell proliferation was evaluated with the bromodeoxyuridine (BrdU) incorporation assay using a colorimetric BrdU kit (Roche, Indianapolis, IN, USA). The BrdU Cell Proliferation Assay is a non-isotopic immunoassay for the quantification of BrdU incorporation into newly synthesised DNA of actively proliferating cells. HDF and HEK were seeded in 96-well culture plates at a density of 5500 and 8000 cells/well in GM-F and GM-K, respectively. The cells were synchronised in the G1/G0 of the cell cycle by overnight incubation in serum-deprived medium. Thereafter, the cells were exposed to three different concentrations of HA matrix (0.05, 0.2 and 1 mg/mL prepared in AM-F with 0.1% FBS) for 24 h and 48 h. The NT cells, maintained in their respective AM, were tested in parallel as an indicative condition of the basal proliferation level of the cultures (negative control). The cells were maintained in GM during the exposure period as a condition for stimulating cell proliferation (positive control). The HDF and HEK were labelled with BrdU following an incubation period of 6 h and 12 h, respectively, at 37 °C. Then, the medium was removed, and the cell proliferation was evaluated by colorimetric immunoassay, using an anti-BrdU antibody for the measurement of the BrdU incorporated during DNA synthesis. The absorbance (370 nm) was measured with the Safire2 plate reader. The cell proliferation was calculated by measuring the difference in optical density (OD) of the tested product with respect to the NT cells.

#### 4.4.4. Cell Migration Assay

The cell migration was assessed using an Oris™ 96-well migration assay kit (Platypus Technologies, Fitchburg, WT, USA) populated with silicone-based cell seeding stoppers, which exclude the cells from attaching to a central zone in each culture well. HDF and HEK were seeded at a final cell density of 15,000 and 20,000 cells/well in GM-F and GM-K, respectively. Once the cells were adhered, the stoppers were removed, revealing 2-mm central cell-free zones (detection zones) into which the cells then migrate. The cells were immediately exposed to three different concentrations of the HA matrix (0.05, 0.2 and 1 mg/mL prepared in AM-F with 1% FBS). In parallel, the cells were exposed to their respective AM (NT—condition) as a basal migration control or to their GM as a migration-stimulating condition. The HDF were incubated for 24 h and the HEK for 48 h. Next, the medium and tested samples were removed, and the cells were stained with a cell-permeant dye, calcein (Sigma-Aldrich), for 30 min at SCC. Fluorescent cell images, as well as the signal intensity (excitation 485 nm, emission 528 nm) of the migrated cells into the detection zone, were obtained with the Cytation 5 cell imaging multi-mode reader (Biotek; Winooski, VT, USA). The cell migration was calculated by measuring the difference in the fluorescence signal intensity of the tested product with respect to the NT cells. The images were analysed using the image processing software Image J Fiji (Bethesda, MD, USA).

#### 4.4.5. Assessment of ECMp Production

The HDF were seeded in a 96-well plate at a density of 6500 cells/well and maintained in GM-F until they reached confluence. Prior to treatment, the cells were starved overnight with a serum-deprived medium (containing 0.1% FBS). Then, the cells were exposed to three different concentrations of the HA matrix (0.05, 0.2 and 1 mg/mL prepared in AM-F with 1% FBS) for 72 h in SCC. The NT, maintained in AM-F during the exposure period, were studied as the basal control. Transforming growth factor beta 1 (TGF-β1) 10 ng/mL; Peprotech, Cranbury, NJ, USA) was used as the reference compound for the induction of ECMp synthesis in the HDF. After treatment, the cells were washed twice with HBSS and fixed with paraformaldehyde (PFA 3%; Sigma-Aldrich) in PBS. The plates were stored at 4 °C until ECMp quantification.

Type I collagen, type III collagen and elastin were quantified by indirect ELISA using the cell layer as the solid phase. Briefly, the plates were incubated with human primary anti-collagen I, anti-collagen III and anti-elastin primary antibodies (Sigma-Aldrich). Then, a biotinylated secondary antibody (Vector Laboratories, Burlingame, CA, USA) and a streptavidin-conjugated horseradish peroxide (streptavidin-HRP, Sigma-Aldrich) were used to improve the detection of the target proteins. Next, the HRP-substrate OPD (SIGMAFAST™ OPD, Sigma-Aldrich) was added and the absorbance (429 nm) was measured with the Safire2 plate reader. In parallel, triplicate assay wells (ELISA control wells) were subjected to the same ELISA processing, but without incubation with the primary antibody, for each protein analysed, to correspondingly determine the degree of basal or nonspecific binding of the biotinylated secondary antibody and streptavidin-HRP system to the cell monolayer. The absorbance values of the assay wells were corrected by subtracting the signal from the ELISA control wells.

The stimulation of each ECMp production was calculated by measuring the difference in the absorbance of the tested product with respect to the NT cells.

#### 4.4.6. Assessment of GAG Production

The stimulative effect of the HA matrix on GAGs synthesis was assessed through quantification of new synthesised GAGs, mainly HA, on HDF after 24 h of exposure to the HA matrix ingredient by a radioactive method.

The HDF were cultured in 24-well culture plates and treated for 24 h with three different concentrations of HA matrix (0.25, 0.5 and 1 mg/mL prepared in AM-F with 1% FBS), then incubated overnight with 25 µCi/mL [3H] glucosamine (glucosamine hydrochloride, D-[6–3H(N)]-, aqueous solution, 1 mCi/mL; PerkinElmer, Waltham, MA, USA). A GAG synthesis promotor agent (TGF-β1 10 ng/mL) was also tested as a reference stimulatory condition. In parallel, the NT, maintained in AM-F during the exposure period, were tested as a basal GAG production control. After treatment and to detect newly synthesised GAG, cell lysis of the test cultures was performed with 4 M guanidine hydrochloride and 1% *w/v* Triton x-100 in Tris-HCl, solution pH 7.4 (Sigma-Aldrich); the resulting lysate was exposed to GAG-specific binding with 2.5% *w/v* Cetylpyridinium (CPC, Sigma-Aldrich) on 3MM-Whatman filter paper, followed by sequential washes with 25 mM sodium sulphate and PBS. Finally, the radioactivity bound to the processed papers, corresponding to the levels of GAGs, were quantified in a liquid scintillation counter (Tri-Carb 2900TR, Packard Bioscience, Meriden, CT, USA) with 3 mL/vial of EcoLite™ Liquid Scintillation Cocktail (MP Biomedicals, Eschwege, Germany). The obtained counts per min (CPM) and disintegration per min (DPM) values were proportional to the newly synthesised GAGs. The stimulation of GAG production was calculated by measuring the difference in the GAG amount of the tested compound with respect to the NT cells.

#### 4.4.7. In Vitro Test of Cell Antioxidant Activity

The antioxidant potential of the HA matrix ingredient was evaluated by quantification of intracellular ROS production on HDF and HEK. The cells were seeded in 96-well plates at a final cell density of 5000 HDF cells/well and 7500 HEK cells/well in their respective GM until confluence. The cells were labelled with the fluorescent probe Carboxy-H_2_DCFDA (Molecular Probes^TM^, Eugene, OR, USA) in a 40 min incubation at SCC. After two washes, three dilutions of the HA matrix (0.05, 0.2 and 1 mg/mL prepared in HBSS) in HBSS were applied. In parallel, a positive control, quercetin in 0.25% DMSO (Sigma-Aldrich), a well-known antioxidant compound, and its vehicle control (0.25% DMSO) were also tested. An “NT” condition was included as an indicator of the levels of ROS production in oxidative stress-induced and non-induced cells. Immediately, the cell cultures were induced to oxidative stress by applying tert-Butyl hydroperoxide (TBHP, Sigma-Aldrich). To prove that these treatments did not exert any inductor effect on the basal levels of ROS, a non-induced-TBHP condition was also tested for each sample, where TBHP was replaced by HBSS. The plates were maintained in the incubator for 4 h in SCC. The fluorescence intensity was measured immediately (at 0 h) and 4 h after oxidative stress insult, with a Safire2 plate reader (excitation and emission wavelengths of 485 and 527 nm, respectively). The antioxidant activity was calculated by measuring the RFU of the tested product with respect to the NT cells.

### 4.5. In Vitro Absorption Study of the HA Matrix

#### 4.5.1. Everted-Gut Sac Assay

The absorption was assessed using the everted-gut sac model. Six male OFA rats weighing approximately 150 g were euthanised by cervical dislocation and the whole small intestine was immediately excised. The intestines were then flushed using Krebs-Henseleit (KB) medium (NaCl 112 mM, KCl 4.7 mM, CaCl_2_ 2.5 mM, KH_2_PO_4_ 1.2 mM, MgSO_4_ 1.2 mM, NaHCO_3_ 25 mM; all reagents by Panreac) and gently everted over an inox rod. After identification of each part (duodenum, jejunum, ileum), segments of approximately 4 cm in length were prepared by securing one end with a silk suture, then filling the sacs with KB solution and finally sealing them with a second tie at the opposite end. One ileum, one duodenum and four jejunum fragments were prepared from each animal. Each filled sac was transferred to a glass tube containing KB medium with 200 µg/mL HA matrix and incubated at 37 °C with aeration. At different incubation times (5, 10, 20 and 30 min), the gut sacs were individually removed (*n* = 8 each time) and gently blotted dry. The sacs were cut open and the contents of each sac were then drained into a separate tube.

The degree of transfer or absorption was calculated per sac, as a percentage, by comparing the internal concentration with the nominal concentration of the incubation medium.

#### 4.5.2. GAG Quantification Assay

The GAG concentration in each sac sample was assayed using a method adapted from Dimethylmethylene Blue Assay, originally described by Farndale et al. (1982) [85]. The method involves the formation of a complex between ionised sulphate and carboxyl groups on the GAG molecules (HA, CS and DS) and the cationic dye 1,9-dimethylmethylene blue (DMMB) [86]. Absorbance of this complex can be read on a spectrophotometer at 535 nm.

The samples of the sac content were immediately analysed after being obtained, together with a calibration curve, in quintuplicate, prepared at increasing concentrations of the HA matrix in a KB solution (range: 2.5–50 µg/mL) and a negative control (KB solution).

Sample analysis was performed by adding 2 mL of DMMB solution to 200 µL of sac content (or calibration curve sample or negative control). After brief vortexing, the mix was placed in a spectrophotometer cuvette and the absorbance was immediately read at 535 nm (Novaspec II Visible Spectrophotometer, Pharmacia Biotech, Uppsala, Sweden). Preparation of the DMMB solution was as follows: 16 mg DMMB (Sigma-Aldrich) were dissolved in 5 mL ethanol (Scharlab, Sentmenat, Barcelona, Spain). Then, 2 g sodium formate (Sigma-Aldrich) and 2 mL formic acid (Across Organics, Geel, Belgium) were added to the DMMB ethanolic solution, and the final volume was completed to 1000 mL with deionised water.

The quantifying method consisted of interpolation along a regression line constructed from the results of the calibration curve (HA matrix concentration vs. OD response). The quantifying procedure was performed using Microsoft Excel software (Microsoft 365).

### 4.6. Statistical Analysis

The in vitro assay data are expressed as the mean value ± standard deviations (SD). A one-way analysis of variance (ANOVA) was performed to examine statistically different expression patterns between groups, followed by a Dunnett’s post hoc test correction for multiple testing. A *p* value of <0.05 was considered statistically significant. The data were analysed using GraphPad Prism (version 7.00 GraphPad software, San Diego, CA, USA).

Statistically significant differences in the absorption degrees among the incubation times and intestinal regions were tested using a non-parametric Kruskal–Wallis test. Pairwise differences between the intestinal parts were tested by a Mann–Whitney test. A *p* < 0.05 was considered statistically significant. The data were analysed using SPSS software for Windows (version 11.5, SPSS Inc., 1999, Chicago, IL, USA).

## 5. Conclusions

This study determines that the HA matrix isolated and purified from rooster comb is composed of HA (67%); sulphated GAGs (12%), including DS and CS; protein (17%), including collagen (10.4%); and water. The HA matrix has shown its potential to stimulate various primordial cellular mechanisms and bioactivities in HDF and HEK that decline with age. The HA matrix is composed of essential components of human skin, supporting its use to treat and prevent skin aging, and contributing to the maintenance of the epidermal function and the normal formation of ECMp and GAGs in the dermis. In addition, the results suggest that the HA matrix may well be absorbed in the intestine. This would signify a potential oral use in addition to its topical use, either as an ingredient of a nutraceutical or a cosmetic product. However, further studies would be needed to corroborate its oral absorption.

Overall, the results of this study suggest that the HA matrix has a variety of potential benefits for the skin, including stimulating cell migration and proliferation, inducing the synthesis of collagen, elastin and GAGs, and protecting against ROS-induced DNA damage and ROS production. These properties may make the HA matrix a useful ingredient in skin care products aimed at improving the appearance of aged skin.

## Figures and Tables

**Table 1:** Physicochemical characterisation of the HA matrix.

Test	Result
Appearance	Off-white hygroscopic powder
Granulometry	100% through 600 µm
pH	7.1
Chlorides	<1%
Nitrogen	6%
Loss on drying	6%

## Data Availability

Not applicable.
